# Alteration of Cellular Behavior and Response to PI3K Pathway Inhibition by Culture in 3D Collagen Gels

**DOI:** 10.1371/journal.pone.0048024

**Published:** 2012-10-23

**Authors:** Brian Fallica, Joseph S. Maffei, Shaun Villa, Guy Makin, Muhammad Zaman

**Affiliations:** 1 Department of Biomedical Engineering, Boston University, Boston, Massachusetts, United States of America; 2 Clinical and Experimental Pharmacology, Paterson Institute for Cancer Research, and School of Cancer and Enabling Sciences, Manchester Cancer Research Centre and Manchester Academic Health Sciences Centre, University of Manchester, Manchester, United Kingdom; 3 Department of Paediatric Oncology, Royal Manchester Children’s Hospital, Manchester, United Kingdom; Northwestern University, United States of America

## Abstract

Most investigations into cancer cell drug response are performed with cells cultured on flat (2D) tissue culture plastic. Emerging research has shown that the presence of a three-dimensional (3D) extracellular matrix (ECM) is critical for normal cell behavior including migration, adhesion, signaling, proliferation and apoptosis. In this study we investigate differences between cancer cell signaling in 2D culture and a 3D ECM, employing real-time, live cell tracking to directly observe U2OS human osteosarcoma and MCF7 human breast cancer cells embedded in type 1 collagen gels. The activation of the important PI3K signaling pathway under these different growth conditions is studied, and the response to inhibition of both PI3K and mTOR with PI103 investigated. Cells grown in 3D gels show reduced proliferation and migration as well as reduced PI3K pathway activation when compared to cells grown in 2D. Our results quantitatively demonstrate that a collagen ECM can protect U2OS cells from PI103. Overall, our data suggests that 3D gels may provide a better medium for investigation of anti-cancer drugs than 2D monolayers, therefore allowing better understanding of cellular response and behavior in native like environments.

## Introduction

Over the last 40 years, 5 year survival rates for cancer patients have risen dramatically, but total death rates have remained stubbornly high [1]. This reflects both our ability to effectively fight primary tumors and our inability to treat secondary or recurring lesions. This clinical failure is mainly due to persistent tumors which arise from resistant populations of cancer cells [2], [3]. The molecular mechanisms of chemoresistance have been extensively studied[4]–[8]; however this has not translated into clinical success. The gene expression profile of tumor cells changes after treatment[9]–[11], leading to a more drug-resistant cell population. This resistant phenotype is initially present in the tumor and is enriched during chemotherapy indicating clonal expansion of the resistant individuals [11]. The ability to understand and predict the characteristics of the resistant cells will help increase our ability to treat chemoresistant tumors.

An important aspect of chemoresistance is the role of the tumor microenvironment. Tumor cells exist within a dynamic 3D environment which is not completely replicated by standard *in vitro* tissue culture techniques. 2D tissue culture plastic and 3D extracellular matrices (ECM), as scaffolds for cell growth, provide the cell with very different biochemical and mechanical environments. Cells growing in 3D matrices have mechano-transducers and ECM adhesion proteins globally expressed on their surfaces; however, cells grown in 2D only interact with a solid substrate on their basal surface [12]. Culturing cells in 2D vs. 3D platforms results in significant changes in cell growth [13], [14], migration [15], [16], morphology [17], [18], and gene expression [12], [14]. The influence of matrix composition and mechanics is critically important when investigating cancer cell drug response [19]. Previous studies have shown that drug response is heavily influenced by ECM mechanics [20], [21]. In addition, patient survival rates can also be linked to the mechanical properties of the surrounding milieu, with a more rigid ECM correlating with poor prognosis [22]. These differences contribute to the observation that 2D *in vitro* systems are poor indicators of *in vivo* processes[23]–[25]. 3D systems therefore provide more clinically relevant results.

Many investigations have shown that ECM interacting proteins can be used as predictors of tumor behavior and patient outcomes[26]–[29], but few have looked at the composition and characteristics of the matrix itself despite indications from mathematical models that ECM density and homogeneity play critical roles in tumor development [30], [31]. This is perhaps due to the difficulty of directly observing and manipulating cells while in biomimetic ECMs *in vitro*.

**Figure 1:**
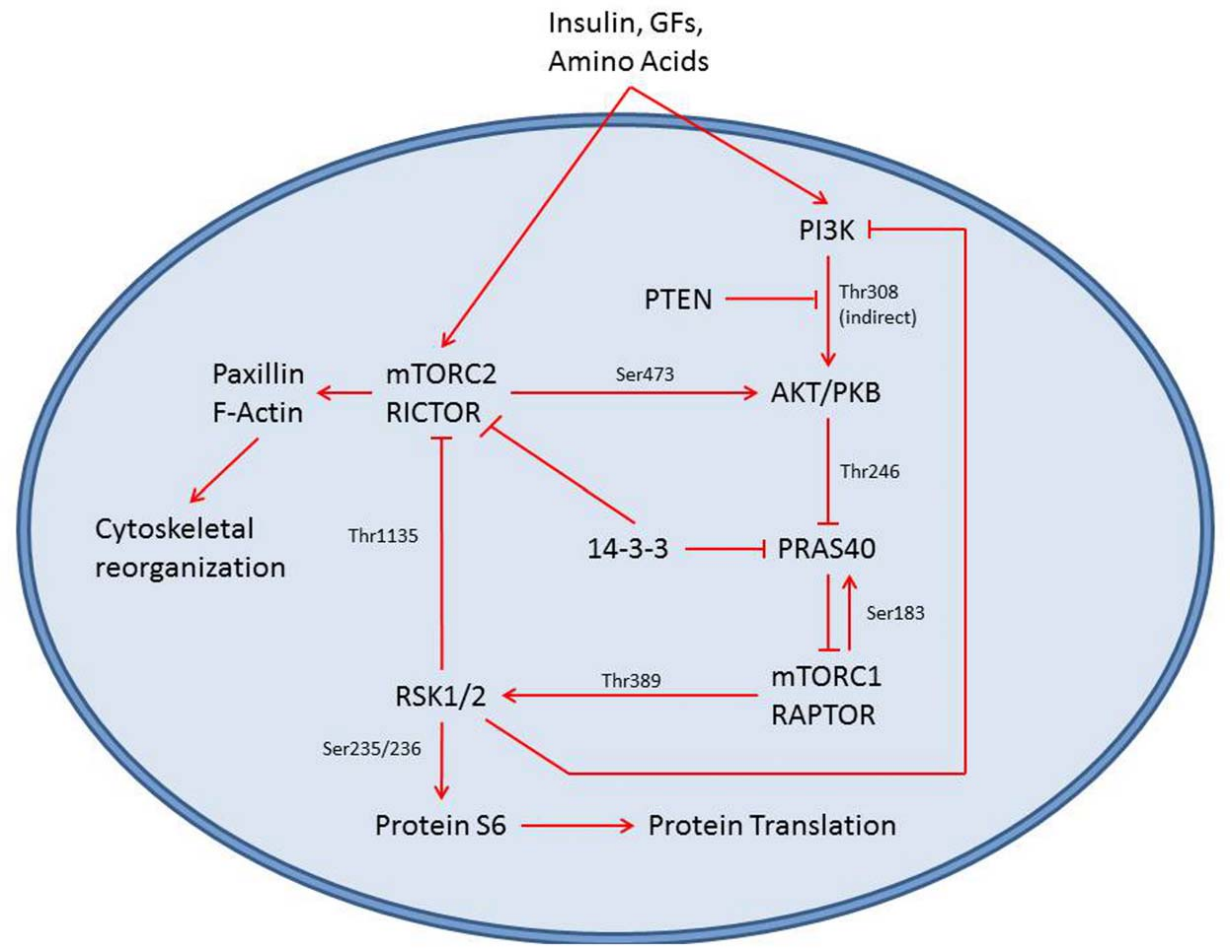
Simplified PI3K/AKT pathway map. In this study we examine the levels of PI3K, mTOR and phosphorylated Protein S6 as indicators of cellular response to PI3K/mTOR inhibition.

The phosphatide inositol 3 kinase (PI3K)/AKT pathway is an important intracellular signaling cascade which affects cell growth, migration, protein expression and survival [32]. Figure 1 shows a simplified version of this signaling pathway. Elevated PI3K activity is observed in many cancers; either due to a constitutively active PI3K mutant or a loss-of-function PTEN mutant, leading to aggressive cell growth and invasion [32]. Several pathway proteins, including PI3K itself and the mammalian target of rapamycin (mTOR) have been investigated as potential targets for new anti-cancer drugs [33]. mTOR is a cellular kinase that is found in two distinct complexes [34]. Both complexes integrate sensors of nutrient and energy levels and regulate downstream cell functions. Increased mTOR Complex 1 (mTORC1) activity results in higher global protein expression, and inappropriate levels of activity have been linked to several kinds of cancer [35]. Phosphorylation of ribosomal protein S6 (pS6) is a downstream indicator of mTORC1 activity [36], whilst phosphorylation of the proline-rich Akt Substrate (PRAS) is an upstream indicator of mTORC1 activity, with PRAS acting to inhibit mTORC1 [37]. mTOR Complex 2 (mTORC2) regulates cytoskeletal organization and turnover as well as AKT phosphorylation. Increased mTORC2 activity leads to the formation of stress fibers and increased cell mobility [38]. Phosphorylation of AKT (pAKT) is a downstream indicator of mTORC2 activity [39]. In this study, we employ the dual kinase inhibitor PI103 [40], [41]. PI103 is a cell-permeable, ATP-competitive inhibitor of both PI3K and mTOR that has been shown to have anti-proliferative affects in various cancer cell lines. PI103 is very potent, with IC_50_ values on the order of 10–100 nM for various isoforms of mTOR and PI3K [40]. It was chosen for this study because U2OS cells express high levels of PI3K/Akt pathway proteins, making them theoretically more susceptible to the effect of PI103. Additionally, PI103 is still being validated as a potential cancer therapy and we believe our 3D culture system is perfectly suited for this type of validation study.

To help understand and quantify the effects of the tumor ECM on cell behavior, we have compared proliferation, migration and protein expression of U2OS osteosarcoma cells between 2D and 3D collagen gels, and investigated the effect of changes in the ECM on inhibition of the PI3K pathway.

## Materials and Methods

### Cell Culture

Experiments were performed on the pediatric osteosarcoma cell line U2OS or breast adenocarcinoma cell line MCF7(ATCC, Manassas, VA). Cells were cultured in complete RPMI media supplemented with 10% fetal calf serum and 1% penicillin-streptomycin solution (10,000 IU/ml penicillin; 10,000 µg/ml streptomycin). Cell cultures were maintained in 2D monolayers in a humidified incubator at 37°C, 5% C0_2_. PI103 [40], a PI3K and mTOR inhibitor was purchased from Cayman Chemical (Ann Arbor, MI).

**Figure 2:**
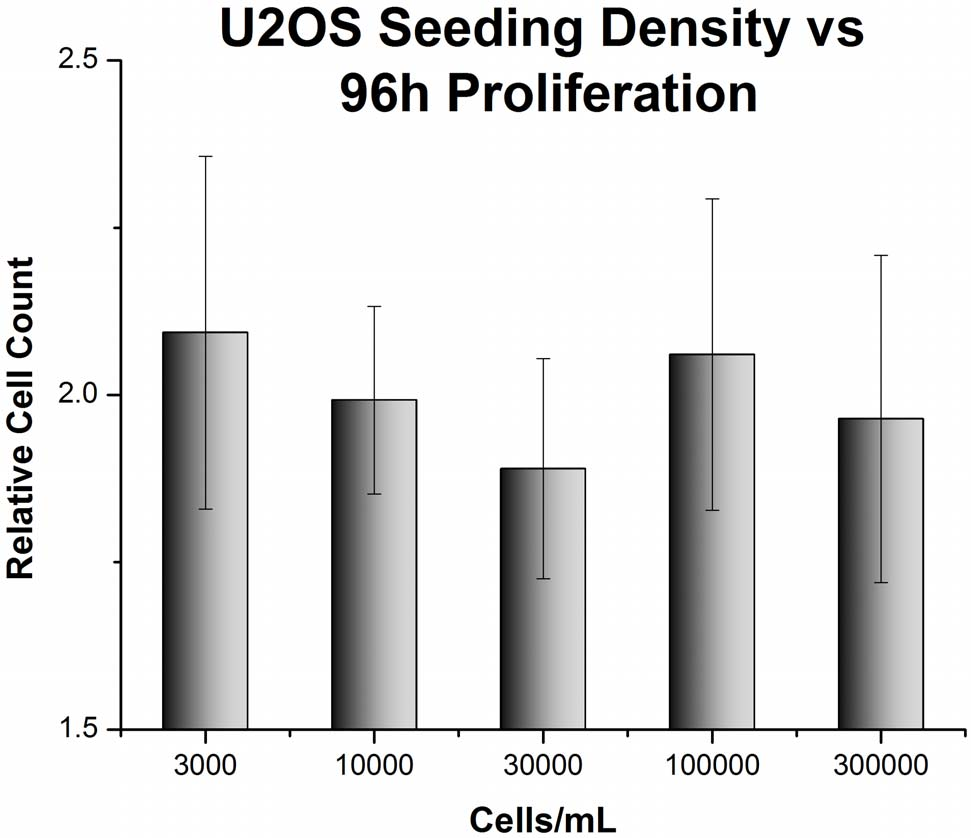
U2OS cell proliferation is unaffected by seeding density. U2OS cells were seeded at various densities in 300 µL collagen gels. Cells were imaged immediately after seeding and 96 hours later. Data is presented as a ratio of the cell population after 96 hours to the cell population at initial seeding.

### Collagen Matrix Preparation

Collagen Type 1 gels were prepared as described previously [42]. Briefly, Collagen Type 1 stock solution (BD Biosciences, San Jose, CA) was diluted to 8.5 mg/ml using 0.02 N Acetic Acid. This solution was diluted to the experimental collagen concentration (3 or 4 mg/ml) by mixing equal volumes of collagen stock solution and neutralizing buffer (100 mM Hepes in 2× PBS, pH 7.3) with PBS. 100 µl of the collagen solution was pipetted into a 96 well plate (MatTek, Ashland, MA) then placed in an incubator (37°C, 5% C0_2_) for 2 hours to allow for complete polymerization. The polymerization time was kept consistent as previous experiments indicated that polymerization time can significantly affect pore size (unpublished data). After polymerization, 100 µl of media was added on top of the gels.

**Figure 3:**
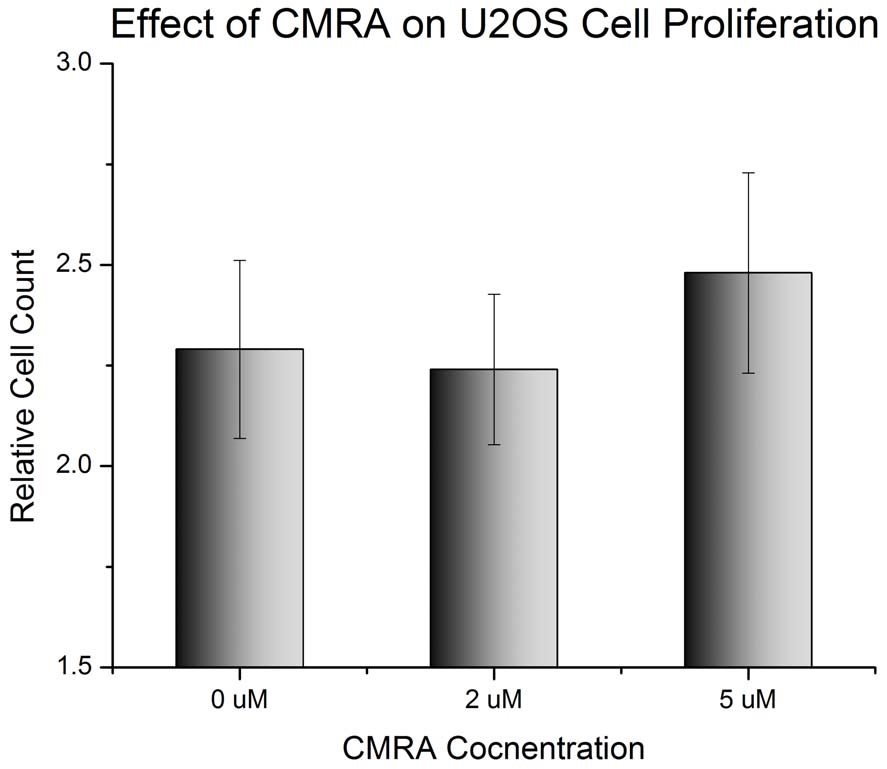
Proliferation of U2OS in 3D collagen gels with different concentration of live cell dye CMRA. Cells were grown in 3 mg/ml collagen gels. Cells were counted immediately after seeding and 96 h post-seeding. Data is presented as a ratio of 96 h data to 0 h data. No significant difference in cell proliferation was observed.

### Cell Tracking Experiments

For cell culture experiments, cells were stained with 5 µM CMRA (Molecular Probes). Cells in 2D experimental groups were plated on tissue culture plastic while cells in 3D conditions were added to the unpolymerized collagen solution to a final concentration of 100,000 cells/ml for the proliferation assay and 30,000 cells/ml for migration trials. The gels were then polymerized as described above, resulting in cell-embedded collagen gels. Media was replaced with fresh media daily. When applicable, experiments were exposed to 250 nM of PI103. For these experiments, PI103 was added to the collagen solutions and the quenching media. Additional drug was added with each subsequent media change to maintain the initial drug concentration.

**Figure 4:**
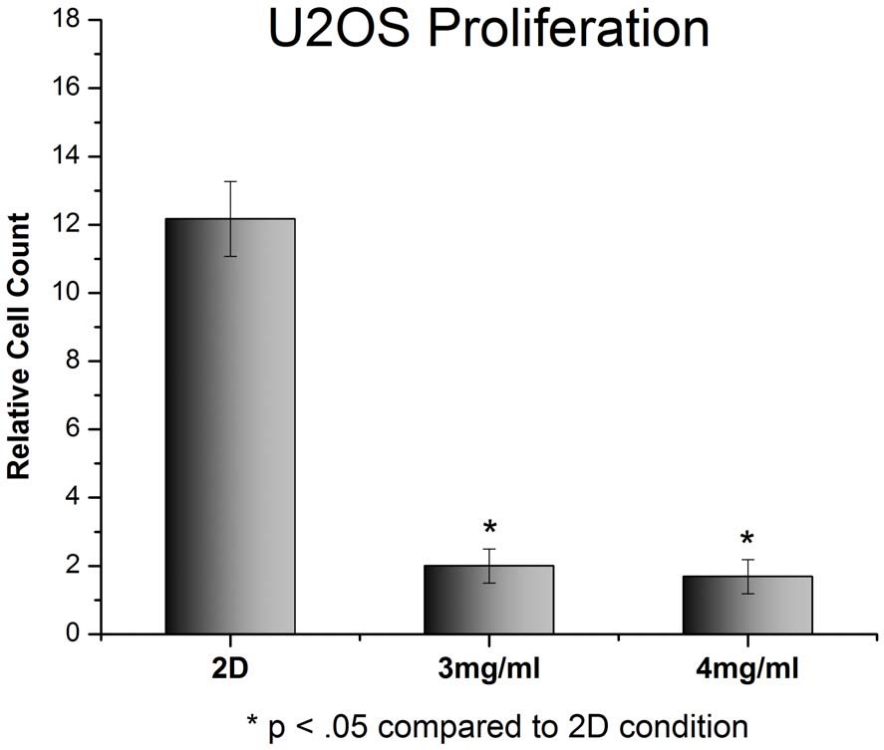
Proliferation of U2OS on standard 2D tissue culture plastic compared to 3D collagen gels. Cells were plated and immediately imaged. 96 hours later the cells were imaged again and the cell population was compared to the initial numbers. Cells in 3D show a dramatic reduction in proliferation, with approximately 1 cell division occurring over the 96 h experiment. Cells in 2D showed much more aggressive growth, with cell doubling occurring every 24–30 hours. (p<.05 between 2D and 3D samples).

**Figure 5:**
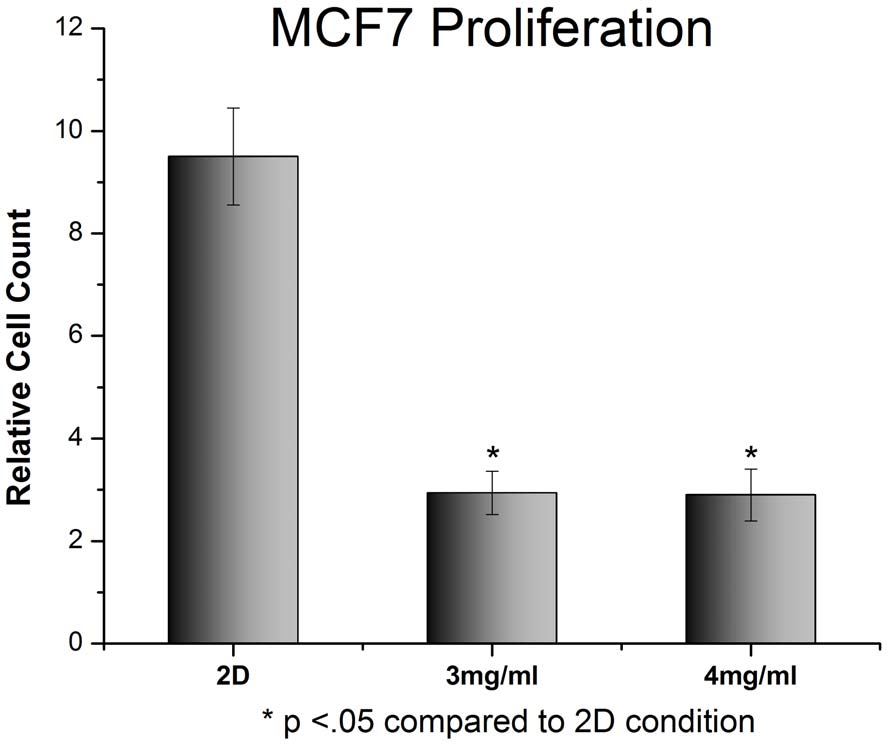
Proliferation of MCF7 cells on standard 2D tissue culture plastic compared to 3D collagen gels. Cells were plated and immediately imaged. 96 hours later the cells were imaged again and the cell population was compared to the initial numbers. Cells in 3D show a dramatic reduction in proliferation. Cells in 2D showed much more aggressive growth, with cell doubling occurring every 30–36 hours. (p<.05 between 2D and 3D samples).

Images were acquired using a DMI600B microscope (Leica, Solms, Germany) and ImagEM EM-CCD Camera (Hamamatsu Photonics, Hamamatsu, Japan) using a spinning disc confocal setup (Yokogawa, Tokyo, Japan). Imaging was done using Micro-Manager 1.4 Software (http://www.micro-manager.org). Confocal images were analyzed using IMARIS 7.3.2 (Bitplane Inc., St. Paul, MN). Cells were analyzed using the spot tracking and surface detection routines. Spot tracking gives the centroid position of each cell at each time point and was used to track cell number and cell movement over time. Surface detection outlined the 3D surface of each cell, and was used for cell size and shape measurements. Cell size was used as an excluding measurement to reject overly small cells from being included in cell number and cell movement measurements.

**Figure 6:**
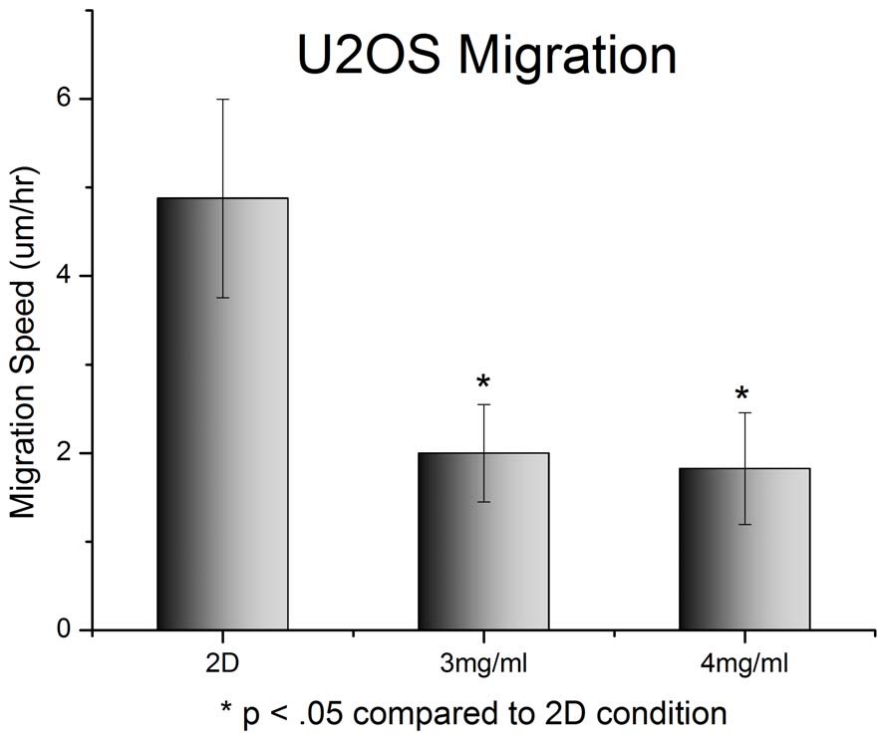
Migration of U2OS cells on standard 2D tissue culture plastic compared to 3D collagen gels. Cells were plated, and then immediately underwent timelapse imaging for 12 hours. Data here is the average speed of the cells over the course of the imaging sequence. Cell tracking was done using Imaris® software suite (Bitplane Inc., St. Paul, MN), with additional data processing done in MatLab®. Cells on tissue culture plastic moved 2.5 times fasters than cells grown in 3D collagen gels (p<.01).

**Figure 7:**
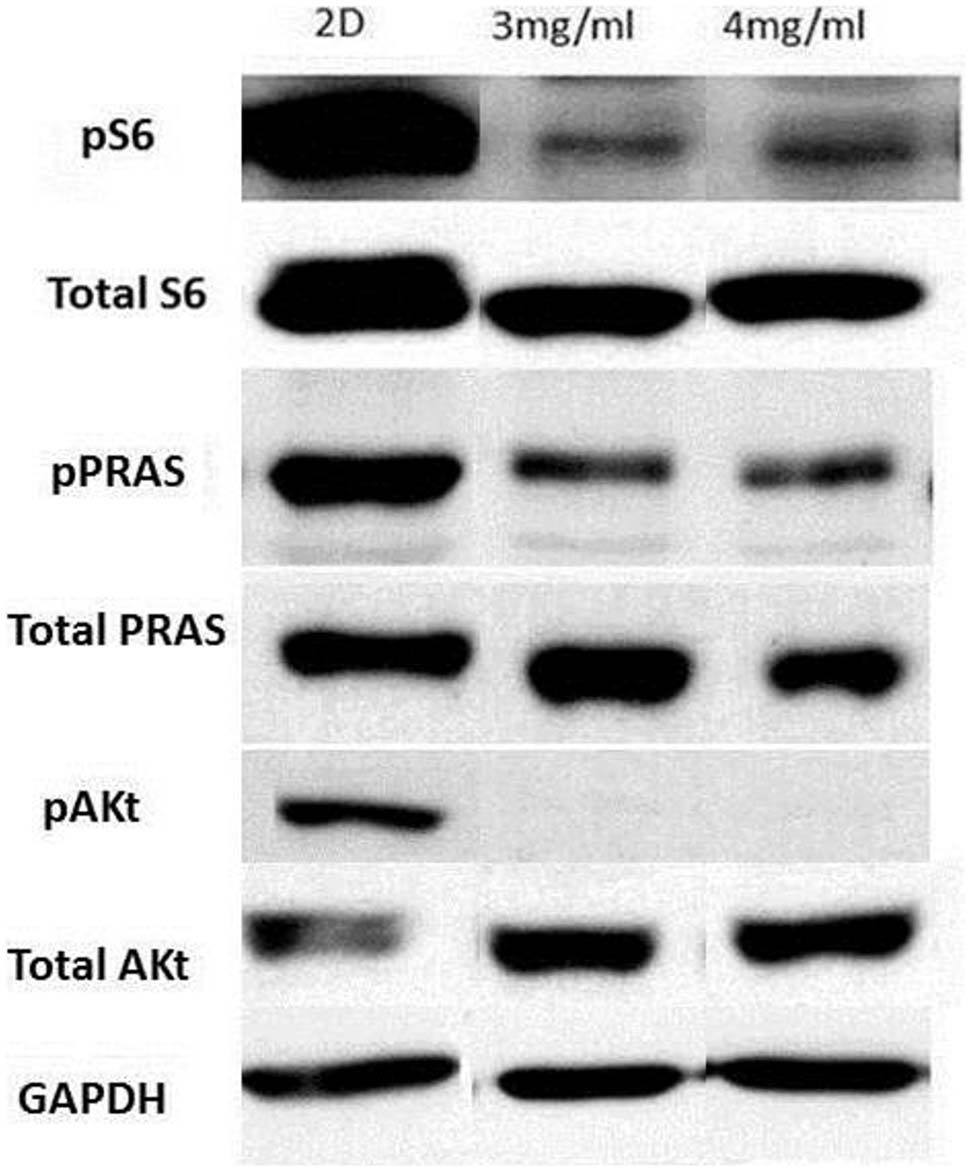
Protein expression blots from U20S cells grown on tissue culture plastic or in 3D collagen gels. Cells were plated, and then harvested 24 hours later. Cell lysates were kept at −80°C if not immediately used. Cells in 3D had undetectable amount of pAkt, indicating a low level of overall pathway activity. This is consistent with the reduction in cell proliferation seen in earlier experiments.

### Western Blot

Cells cultured in 3D collagen were released from collagen gels by incubation with collagenase (100 U/mg/ml, Gibco) at 37°C for one hour. Cells harvested from standard tissue culture plastic were also treated with collagenase to ensure this treatment did not affect results. Liberated cell suspensions were normalized by cell count and lysed in lysis buffer (10 mM Tris, 10 mM NaCl, 1 mM EDTA, 1% v/v triton x100, 10% v/v glycerol, 0.01% w/v SDS, 0.05% w/v deoxycholate, buffered to pH 7.4) for 15 minutes on ice and centrifuged for 10 min at 15,000×g. Whole cell lysates were then subjected to 10% SDS-PAGE followed by electrotransfer onto PVDF membranes. Membranes were blocked overnight at 4°C in 5% non-fat dry milk (BioRad) in Tris buffered saline with 0.1% Tween-20 (TBST, 20 mM Tris pH 7.6, 135 mM NaCl) before primary antibody incubation in 0.5% non-fat dry milk in TBST (Santa Cruz) for one hour at room temperature. After washing in TBST, membranes were incubated in secondary (horseradish-peroxidase conjugated) antibodies (AbCam) in TBST for one hour at room temperature. Membranes were visualized with Pierce ECL substrate (Thermo Scientific). All protein bands were normalized to GAPDH content and analyzed via densitometry using ImageJ.

**Figure 8:**
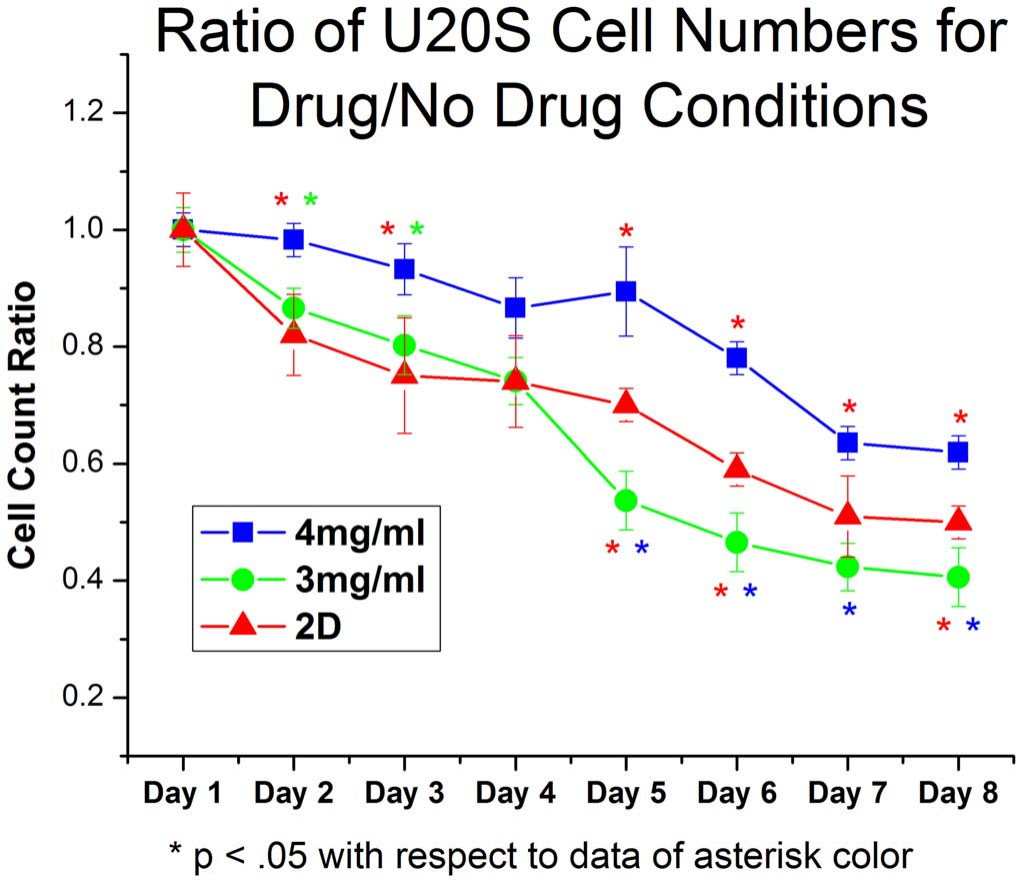
Ratio of U20S cell number in samples treated with PI103 to samples left untreated. Data here is presented as a ratio of cell count in drug treated sample to untreated controls. Cells in 4 mg/ml collagen gels had a 90% cell survival rate after 8 days of drug treatment. Cells were more sensitive to the effects of the drug on 2D tissue culture plastic and in 3 mg/ml gels. Interestingly, cells showed similar behavior in 3 mg/ml and 4 mg/ml gels when untreated, but had a markedly different response to PI103. All Day 8 data points are significantly different (p<.05).

## Results

In order to investigate the effects of the ECM on cell behavior the proliferation, migration, protein expression and response to PI3K inhibition of U2OS and MCF7 cells on tissue culture plastic and in Type 1 collagen gels of different collagen concentrations was investigated. U2OS cells were chosen due to their high native PI3K/Akt pathway activity [43], giving us an attractive drug target. Additionally, U2OS cells grow well in our collagen gels and on tissue culture plastic, making them very easy to work with. MCF7 cells were chosen due to their wide use in *in vitro* experiments. The cells are also very robust and react well to being embedded in collagen gels.

**Figure 9:**
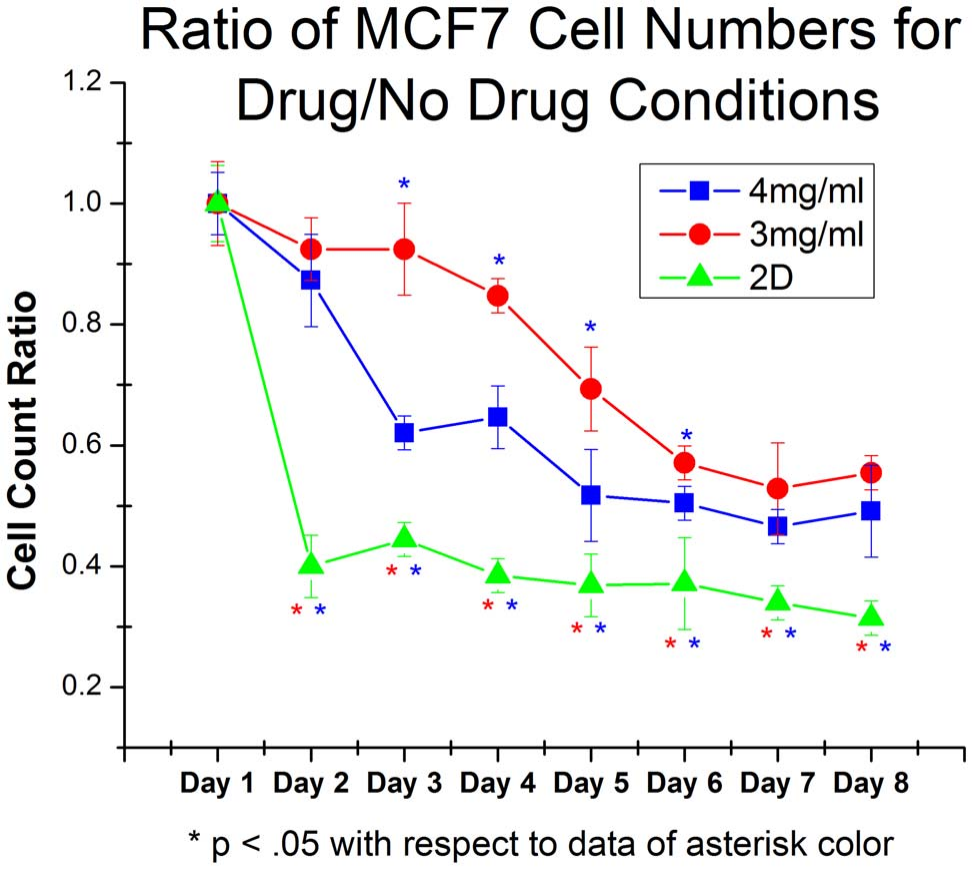
Ratio of MCF7 cell number in samples treated with PI103 to samples left untreated. Data here is presented as a ratio of cell count in drug treated sample to untreated controls. As in figure 8, data is presented as a ratio of cell counts in the drug treated and untreated samples. Similar to U2OS cells, MCF7 cell survived very well in 4 mg/ml collagen gels when compared to cells in 2D. Unlike U2OS cells, MCF7 cells in 3 mg/ml collagen gels survived equally as well as cells in stiffer gels. Both of these conditions yielded cells with higher drug resistance than cells grown in monolayer. Although this behavior is not as complex as observed in U2OS cells, it highlights the difference between 2D and 3D culture platforms.

**Figure 10:**
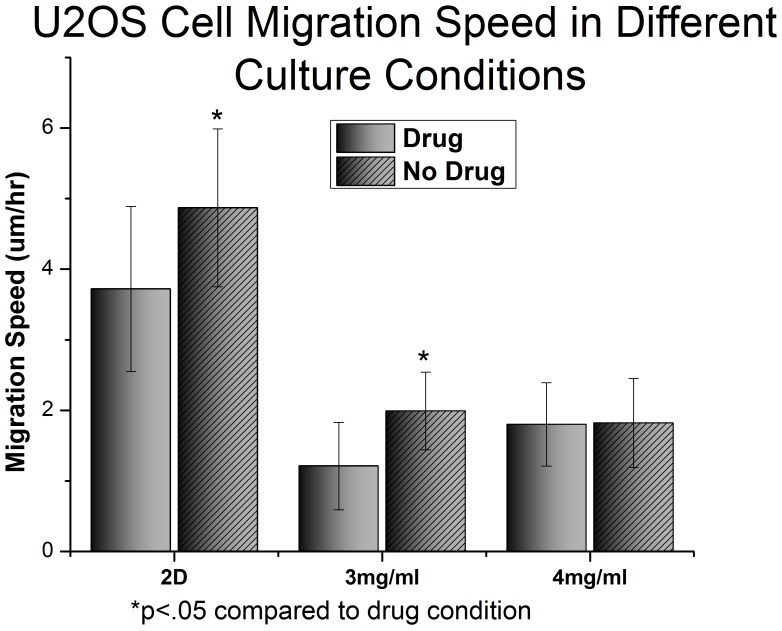
Migration of U2OS cells over 6 hours in different culture conditions. Cells were stained with CMRA whole cell stain prior to being embedded in collagen. Cells were tracked by imaging the gels every 15 minutes for 6 hours. Cell speed was calculated using a 3D displacement algorithm performed on cell centroid location data from Imaris®.

Because the 3D culture conditions introduce inherent differences in local cell density, the growth of cells seeded at different densities within collagen gels was analyzed. As can be seen in Figure 2 a 100 fold variation in the initial seeding density, from 3,000 to 300,000 cells/ml has no effect on the proliferation of U2OS cells in 3D gels containing 3 mg/ml collagen over a 96 hour time course; at each seeding density the U2OS cells roughly double over the 96 hrs of the experiment. Thus over this range of cell densities there is no effect of seeding density on the growth of U2OS cells in 3D collagen matrices. Similar results were obtained for the MCF7 cell line (data not shown). A seeding density of 100,000 cells/ml was chosen for subsequent 3D samples. For experiments in 2D, the seeding density was chosen so that cells would not reach confluence prior to the end of the experiment. U2OS and MCF7 cells become confluent at approximately 30,000 cells/cm^2^, and were therefore seeded at 1000 cells/cm^2^.

**Figure 11:**
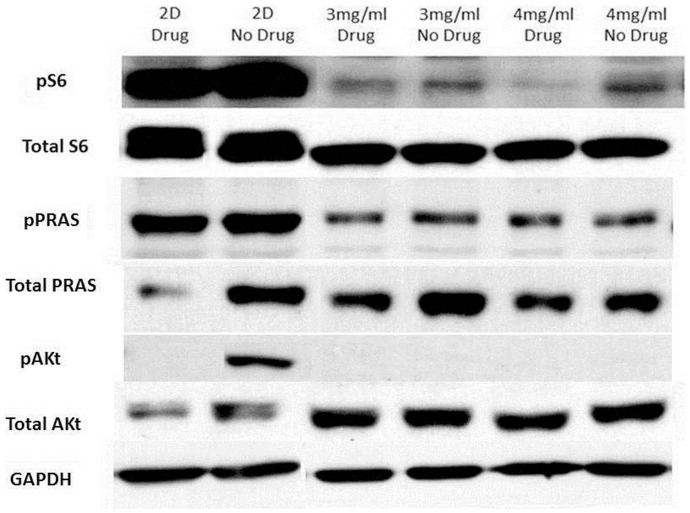
Western Blots for protein S6, PRAS, Akt and GAPDH in U2OS cells on different substrates with and without drug treatment. Cells were plated, and then harvested 24 hours later. Cell lysates were kept at −80°C if not immediately used. Drug treatment reduced levels of pS6 in all samples, consistent with the observed increase in cell doubling time. Paradoxically, this reduction was most pronounced in cells grown in 4 mg/ml despite these cells showing the highest proliferative resistance to the effects of PI103.

Next, the effect of the live cell dye on the proliferation of U2OS and MCF7 cells in 3D collagen gels was investigated. The CMRA fluorophore has been used previously without effect on cell behavior [44], but this was not in 3D culture conditions. U2OS cells grown in 3 mg/ml collagen type 1 gels were stained with 0 µM, 2 µM or 5 µM CMRA and cell number was monitored over 96 h. Figure 3 shows that CMRA did not affect cell proliferation in 3 mg/ml type I collagen gels. The same control was performed on MCF7 cells, which showed a similar insensitivity to the presence of CMRA. Differences in the behavior of U2OS and MCF7 cells between 2D and 3D culture conditions cannot be due to the inclusion of the dye.

### Presence of a 3D ECM Affects Cell Behavior

The proliferation of U2OS and MCF7 cells was examined in type I collagen gels of varying densities. U2OS cells proliferated significantly slower in 3D collagen gels than in 2D monolayer (Figure 4). Cells grown as 2D monolayers on tissue culture plastic had doubling time of 24–30 hours, whilst cells grown in either 3 mg/ml or 4 mg/ml type I collagen gels only doubled once over the 96 hour experiment. Similar results were observed in MCF7 cells (Figure 5), although MCF7 cells grew slower in 2D and faster in 3D compared to U2OS cells.

Next the migration speeds of U2OS cells in 3D collagen gels and on tissue culture plastic were compared. U2OS cells moved significantly faster on 2D tissue culture plastic than in either 3 mg/ml or 4 mg/ml type I collagen gels (5 µm/hr in 2D compared to 2 µm/hr in 3D; p<10^−5^) (Figure 6) This is consistent with previous studies, which have shown that migration speed of cancer cells [45] and fibroblasts [46] is severely inhibited in 3D conditions. The same experiment was attempted on MCF7 cells, but these cells showed negligible (<500 nm/hr) movement in 3D. This also made subsequent investigations into the effect of PI103 on MCF7 cell migration unnecessary since there is no movement for the drug to inhibit.

This evaluation of the proliferation and migration of U2OS and MCF7 cells clearly shows that these cells behave very differently in 3D collagen gels than on flat 2D substrates. Because the PI3K pathway is critical to multiple cellular processes, the levels of key proteins in the PI3K/Akt pathway were compared between U2OS cells grown in 2D and 3D (Figure 7). U2OS cells growing in both 3 mg/ml and 4 mg/ml type I collagen gels showed considerably lower levels of phosphoS6 and phosphoPRAS than when grown in 2D monolayer, and these differences were far greater than any differences observed in levels of total S6 and total PRAS between these conditions. PhosphoAkt (pAkt) was undetectable in cells grown in 3D despite there being more total Akt in U2OS cells grown in 3 mg/ml and 4 mg/ml type I collagen gels than in 2D monolayer. Overall the reduction in levels of these phosphorylated proteins in U2OS cells growing in 3 mg/ml and 4 mg/ml type I collagen gels strongly suggest that the PI3K/mTOR pathway is much less active under these conditions, which would be in keeping with the observed reduction in cellular proliferation and motility in 3D gels.

### Collagen Content and Dimensionality Affect Efficacy of PI103 Treatment

Culturing U2OS and MCF7 cells in 3D collagen gels produces very different behavior than on standard tissue culture plastic. Further, in U2OS cells the move to a 3D culture platform yields very different levels of activity in the important PI3K/MTOR pathway. How does this difference in behavior and PI3K pathway activation affect the response of U2OS cells to pharmacological inhibition of the PI3K/mTOR pathway? As can be seen from Figure 8, the response of U2OS cells to the pan PI3K and mTOR inhibitor PI103 is more complex than the differences in cell behavior and PI3K pathway activation might suggest. The proliferation of U2OS cells growing as a 2D monolayer is clearly reduced by PI103 such that by day 8 of treatment there are only 50% as many cells present as in the untreated control. However, although the effect of PI103 on cell number is less on U2OS cells growing in 4 mg/ml type I collagen gels, as would be anticipated from their reduced proliferation and reduced pathway activity, the response of U2OS cells growing in 3 mg/ml type I collagen gels is very different. Despite there being no appreciable difference in the activation status of the PI3K pathway between U2OS cells grown in 3 mg/ml and 4 mg/ml type I collagen gels (Figure 7), in U2OS cells growing in 3 mg/ml type I collagen, PI3K pathway inhibition by PI103 produces a significantly greater reduction in proliferation than in either 2D or 4 mg/ml type I collagen; after 8 days of treatment there are only 40% as many cells as in the untreated controls.

We performed the same experiment on MCF7 cells to show that these results can be obtained from multiple cell lines (Figure 9). After 8 days, cells in 3D (both 3 mg/ml and 4 mg/ml gels) showed very similar survival, whereas cells in 2D were significantly more sensitive to the anti-proliferative effects of PI103. Interestingly, at early time points there was a significant difference between the 3D samples, but this discrepancy was gone after 8 days. We plan on exploring protein expression in MCF7 cells in these conditions in future studies.

Given the reduction in motility in U2OS cells grown in type I collagen gels in comparison with that in 2D monolayer the effect of PI3K pathway inhibition on cellular motility in these different conditions was also investigated. As can be seen in Figure 10, U2OS cells growing in 2D monolayers are significantly less motile after treatment with PI103 than when untreated, and there is no effect of PI103 on the motility of U2OS cells growing in 4 mg/ml type I collagen gels. However U2OS cells growing in 3 mg/ml collagen gels showed significant reduction in motility after treatment with PI103. Under these conditions there was a 39% reduction in cell speed after PI103 treatment in comparison with a 24% reduction in cell speed in U2OS cells growing in 2D monolayer. Thus not only does culturing U2OS cells in 3D collagen gels have significant effects upon their proliferation and motility, and significantly reduce the activation of the PI3K pathway, but it also alters the way in which these cells respond to pharmacological inhibition of this pathway. However the situation is more complex as the concentration of collagen within the gel also has a significant effect on the response of U2OS cells to PI3K pathway inhibition.

### PI103 Reduces Expression of PI3K Pathway Proteins in 2D and 3D

In an attempt to explain the observed difference in drug sensitivity between the two different collagen concentrations the effect of PI103 treatment on the levels of various phosphoproteins in the PI3K pathway was assessed. As can be seen from Figure 11, PI103 treatment reduces levels of pAkt to undetectable in U2OS cells growing in 2D monolayer. There is no appreciable effect of PI103 on levels of phospoPRAS in either 2D or 3D conditions. PI103 reduces levels of pS6 in both 2D and both 3D conditions. Thus there is no clear difference in PI3K pathway phosphoprotein responses to PI103 between U2OS cells growing in 3 mg/ml type I collagen gels and those growing in 4 mg/ml collagen gels, despite the very clear differences in anti-proliferative and anti-motility effects of this agent under these two conditions.

## Discussion

Our study provides quantitative understanding of the role of ECM in altering cellular behavior, in particular the response of PI3K pathway, in 2D and 3D environments. Our data indicates that the presence of a three-dimensional ECM significantly affects the proliferation and motility of U2OS and MCF7 cells and is consistent with emerging research into the role of ECM in cell behavior [47]. This study, along with several others[13], [23], [48]–[50], indicates that the 2D cell culture paradigm is limited in its capacity to provide detailed understanding of cellular behavior and that the incorporation of 3D culture techniques would be of considerable benefit for a comprehensive understanding of cellular behavior. Type 1 collagen gels are an attractive platform for future *in vitro* investigations of cell behavior. The gels are easy to create, inexpensive and are conducive to live cell manipulation and imaging. The gels are also customizable, with the ability to control pore size, ligand density and stiffness by either changing the concentration of collagen [16], [18], [47] or by introducing chemical crosslinking compounds to alter the gel structure [48], [51]. All of these ECM characteristics are tightly controlled *in vivo* and can contribute to many kinds of pathologies.

Our study demonstrates that the presence of a 3D ECM is sufficient to alter the activation of the PI3K/Akt pathway in U2OS cells. This reduced activation was consistent with an observed reduction in cellular proliferation and migration speed in cells growing in 3D matrix. Future investigations are needed to understand how a mechano-chemical signal from the ECM is transduced to intracellular signaling cascades.

Previous work has shown that the presence of ECM proteins is sufficient to confer resistance to cancer cells against anti-tumor agents [52]. Our data supports this observation, but also shows that the drug response is affected by ECM properties including mechanical stiffness and protein content. Higher collagen concentrations resulted in a more resistant population in U2OS cells, which is in agreement with the observation that increased collagen 1 content in certain tumors is associated with poor patient survival [22]. Further study is needed to understand why the stiffer gels conferred a selective advantage to the U2OS cells. One hypothesis is that the cells in 4 mg/ml gels were able to activate an alternate survival pathway in order to circumvent the drug’s inhibition of key PI3K/Akt pathway proteins. A likely candidate for this alternative pathway is the MAPK pathway[53]–[56]. In MCF7 cells, the presence of a 3D collagen matrix was sufficient to confer a resistance to the anti-proliferative effect of PI103. Although it appears the collagen content of these gels may have a role in how these cells respond to drug over time, at the end of our 8 days experiment there was no significant difference between cells in gels with different collagen content. We did see a significant difference between MCF7 cell in 2D and 3D, further highlighting the potential use of 3D culture techniques as a more biomimetic drug validation platform.

The fact that the ECM is sufficient to alter cell behavior, protein expression and drug response must be taken into account when designing and executing drug candidate validation studies and clinical trials. Currently, the vast majority of novel cancer therapeutics are screened for their efficacy against well-established cancer cell lines on high throughput, plate-based 2D systems [57]. This protocol completely ignores the native tumor mechano-environment as well as the effects of dimensionality and ligand binding which can also play a major role in drug efficacy. Our results also suggest that simply switching to 3D culture techniques is not enough to ensure a more robust and predictive model. Simply varying collagen concentration in our gels was sufficient to drastically alter cell behavior in one cell line, and this sensitivity to matrix properties must be considered when designing 3D culture systems. As we further our understanding of this relationship, it could be used to predict how tumors respond to different therapies. This type of predictive knowledge is immensely valuable to clinicians, who could use it to intelligently deploy the wide range of drugs currently available to them.
